# The Possible Factors Correlated with The Higher Risk of Getting Infected by COVID-19 in Emergency Medical Technicians; A Case-Control Study

**DOI:** 10.30476/BEAT.2021.89713

**Published:** 2021-04

**Authors:** Mostafa Sadeghi, Peyman Saberian, Parisa Hasani-Sharamin, Fatemeh Dadashi, Sepideh Babaniamansour, Ehsan Aliniagerdroudbari

**Affiliations:** 1 *Prehospital and Hospital Emergency Research Center, Tehran University of Medical Sciences, Tehran, Iran*; 2 *Department of Anesthesiology and Critical Care, Shariati Hospital, Tehran University of Medical Sciences, Tehran, Iran*; 3 *Department of Anesthesiology, Imam Khomeini Hospital Complex, Tehran University of Medical Sciences, Tehran, Iran*; 4 *Tehran Emergency Medical Service Center, Tehran, Iran*; 5 *School of Medicine, Islamic Azad University of Medical Sciences, Tehran, Iran *; 6 *School of Medicine, Shahid Beheshti University of Medical Sciences, Tehran, Iran*

**Keywords:** COVID-19, Emergency medical technician, Personal protective equipment

## Abstract

**Objective:**

To assess the possible factors associated with increasing risk of COVID-19 among EMTs.

**Methods:**

This study was a case-control study conducted in Tehran, Iran. Case group was consisted of confirmed COVID-19 EMTs based on the results of reverse transcriptase polymerase chain reaction and/or lung computed tomography scan. Healthy EMTs were randomly selected as control group. Patients were asked to fill out a checklist including demographic data, data related to the work situation (such as number of missions and type of mask and cloth) and PPE precautions.

**Results:**

Sixty-eight patients and 148 healthy persons took part in this study as case and control group, respectively. Having two EMTs involved directly in taking care of patients (*p*<0.001) and working with a confirmed case teammate (*p*<0.001), considering the precautions such as seal check after wearing the mask (*p*=0.015), covering the hair with a medical hat (*p*<0.001), not using personal items despite protective clothing (*p*<0.001), and avoiding contact with the outer surface of clothing while removing (*p*<0.001) had significant difference in two groups.

**Conclusion:**

We found that the type and method of use of PPE were correlated with the increasing risk of COVID-19 in EMTs. Also, we found that when two EMTs were involved directly in taking care of the patients, and those who worked with a confirmed case teammate, more frequently affected.

## Introduction

Severe acute respiratory syndrome coronavirus 2 (SARS-CoV-2) caused the ongoing coronavirus disease-19 (COVID-19) pandemic, became the most important challenge for the health care settings worldwide [1-3]. The experience from the previous pandemic of coronaviridae family showed that frontline health care workers (HCWs) were among the high risk persons due to close contact with the infected cases, touching the contaminated surfaces and performing the high risk procedures in airway management [4-8]. Emergency medical technicians (EMTs) are among HCWs that may even be at higher risk, as they regularly face with both symptomatic and asymptomatic cases [9, 10]. So EMTs have a high priority for protecting against COVID-19 and also they need additional transmission-based precautions [4, 6, 11, 12]. The World Health Organization (WHO) claimed that HCWs including EMTs, have the rights to access adequate personal protective equipment (PPE) supplies, training about infection control, personal hygiene, waste management, and etc. to decrease the occupational risk of transmission [13, 14]. EMTs were recommended to use PPE like the surgical or N95 mask, gowns and shield in a correct order, and also being trained about it. Various studies showed that factors such as using adequate supplies of PPE, adequate training and education about using the PPE played an important role in terms of decreasing the risk of transmission [14]. The goal of this study was to assess the role of possible factors associated with increasing risk of COVID-19 among EMTs.

## Methods

*Study Design*

This study was a case-control study conducted in Tehran, Iran. The implementation of the project was approved by the ethics committee of Tehran University of Medical Sciences (Code: IR.TUMS.VCR.REC.1399.323); and required permission from EMS center was also received. To maintain the principles of confidentiality, all information was analyzed and reported anonymously. Participants entered the study with informed consent.

Study Population

During 18^th^ February to 20^th^ April 2020, all confirmed COVID-19 EMTs (without any sex or age limit) based on the results of reverse transcriptase polymerase chain reaction (RT-PCR) and/or lung computed tomography (CT) scan were considered as the case group. During the considered study period, we have 70 confirmed COVID-19 EMTs, and the sample size in control group was considered as twice the case group. The EMTs in control group were selected based-on systematic simple random method from list of healthy EMTs during the study period. For this purpose, each EMT was identified by a unique code, then the first subject was selected based on a random number generated by computer software and the rest of the subjects were selected in a fixed sequence until the sample size was completed.

Data Gathering

All the participants underwent phone interview and the data were collected in a checklist of two sections. This checklist was researcher made and the face validity and also clarity, relevancy and comprehensiveness assessed with research team. First section was about demographic data and basic information including age, gender, weight, height, working simultaneously in another healthcare center other than the EMS, history of at least one chronic disease, optional use of any drug or medical supplement as prophylaxis and smoking status. The participants were also asked about the number of EMTs involved directly in taking care of patients, a history of working with an infected teammate whose infection had not been confirmed and before his quarantine, a history of having a confirmed case as their cohabitant, the number of missions, and the daily work-time. The type of face masks including S (the surgical masks), N (the N95 masks), S & N, and E (the elastomeric respirator) used before entering the place where had a possible or confirmed case, the type of cloth including U (uniform), I (disposable, one-piece, waterproof protective cloth), G (gown), NW (disposable, one-piece, non-waterproof protective cloth), I-NW, G-NW, and I-G type used before entering the place where had a possible or confirmed case, and general precautions during their daily life (washing hands, social distancing, using masks in crowded places and etc.).

The second section was about evaluating PPE use precautions and assessing the risk of infection among EMTs who had a history of exposure to a possible or confirmed COVID-19 case. The participants were asked to fill out a seven-part checklist consisting of 43 questions, in which each item had zero (no), one (to some extent) or two (yes) score. The whole phone interview with each person took about 15-20 minutes. This checklist was about access to PPEs, patients’ care precautions, following the PPE precautions, doffing PPE correctly, precautions during the patient transfer, knowledge about disinfection process, and post-exposure measures that was prepared by the WHO for such assessment [7, 15]. The information of participants in the case group was based on the events that occurred two months before their diagnosis, and the information of participants in the control group was based on the events that occurred two months before the interview. The participants blinded from aim of the study. In order to being the same precision and accuracy in collecting data in case and control groups, the interviewer was also kept unaware of whether the interviewee was infected or not.

Statistical Analysis

All the data were analyzed using SPSS version 24.0 (SPSS Inc., Chicago, IL, USA). The quantitative variables were described using the mean±SD and the qualitative variables were described using the frequency and percentage of the data. The relation between the categorical variables was examined using the Chi-square test and Fisher’s exact test. The relationship between quantitative variables among the groups was examined using the independent sample T-test. *p*-value<0.05 was considered statistically significant.

## Results

Among 1600 EMTs working for Tehran EMS during study period, there were 70 confirmed cases of COVID-19 during the study period. Four of them were excluded because they did not answer the phone call. Sixty-six confirmed cases and 148 healthy EMTs took part in this study (all were male). The baseline information of the participants is shown in Table 1. Among all participants in the case group, 64 (97%) cases were quarantined at home (with a mean of 13.35±6.49 and a minimum and maximum of 3 and 32 days). Only 17 (11.4%) cases were admitted to the hospital with a mean duration of 0.52±2.02 days of hospital admission. The frequency of participants in the case group who underwent RT-PCR and lung CT scan were 31 (47%) and 59 (89.4%), respectively. Apparently, some EMTs underwent only one diagnostic test and others underwent two of them.

**Table 1:** Demographic and baseline data of two study groups

**Variables**		**Case group (n=66)**	**Control group (n=148)**	***p*** **-value** ^a^
Overtime working as HCW^b^s other than EMT^c^	No	50 (75.8)	97 (65.5)	0.361
	Yes	15 (22.7)	40 (27)	
Optional use of any drug or medical supplement as prophylaxis	No	43 (65.2)	71 (48)	0.194
	Yes	23 (34.8)	57 (38.5)	
History of at least one chronic disease	No	54 (81.8)	96 (64.9)	0.159
	Yes	10 (15.2)	9 (6.1)	
Smokers	No	64 (97)	128 (86.5)	0.509
	Yes	2 (3)	9 (6.1)	
Number of EMTs involved directly in taking care of patients	One	26 (39.4)	122 (82.4)	<0.001
	Two	36 (54.5)	26 (17.6)	
Working with a confirmed case teammate who did not know about the self-infection	Yes	24 (36.4)	23 (15.5)	<0.001
	No	13 (19.7)	62 (41.9)	
Age (year)		35.72±7.84	35.09±6.76	0.550
BMI^d^ (kg/m^2^)		26.39±3.22	25.69±3.13	0.168
Number of missions during last two months		157.88±97.1	210.8±112.61	0.002
Last Two Months working hours		470.09±124.84	548.87±155.77	<0.001
Mean of daily work time (hour)		9.16±2.77	9.15±2.6	0.982

^a^ *p*-value refers to the relationship of each variable with two groups. Data was described using frequency(percent) or mean±SD. ^b^HCW: Health Care Worker; ^c^EMT: Emergency Medical Technicians; ^d^BMI: Body Mass Index.

The frequency of participants in the case group having a confirmed COVID-19 person as their cohabitant before getting the infection was reported by 6(9.1%) EMTs. Working simultaneously in another healthcare center other than the EMS, the optional use of any drug or medical supplement as prophylaxis, smoking status, age, BMI, the daily work-time and the daily missions’ time had no significant difference among the two groups (*p*>0.05). The case group was twice as likely as the control group to have the history of at least one chronic disease, and the presence of chronic disease increased the risk of infection 1.98 times higher (95%CI: 0.76 to 5.16) but the difference was not statistically significant (*p*=0.159). Using any drug or medical supplement as prophylaxis in the control group was more frequent than the case group (44.5% vs 34.8%), but the difference was not statistically significant (*p*=0.194).

The case group mostly had two EMTs involved directly in taking care of patients which significantly increased the risk of infection 6.5-times higher (95% CI: 3.36 to 12.55, *p*<0.001). Working with a confirmed case teammate whose infection had not been confirmed at the moment and before his quarantine or isolation significantly increased the risk of infection 4.98 times higher (95% CI: 2.187 to 11.38, *p*<0.001).

Table 2 presents the PPE use precautions and self-care implementation in dealing with COVID-19 patients by participated EMTs. General precautions of daily life and the type of face masks used before entering the place where had a COVID-19 case had no significant difference among the two groups (*p*=0.552). The percentage of using the S-type face mask was significantly higher and using the N-type face mask was significantly lower in the case group before entering the place had a confirmed COVID-19 case (*p*=0.865). The percentage of using the I, G, and NW-type cloths before entering the place where had a suspected COVID-19 patient was significantly higher in the case group. The I-type cloth was the most common cloth used in the control group and the NW-type cloth was the most common cloth used in the case group before entering the place where had a confirmed COVID-19 patient (*p*<0.05). Precautions such as seal check after wearing the mask (*p*=0.015), covering the hair with a medical hat (*p*<0.001), not using personal items such as jewelry and mobile phone despite protective clothing (*p*<0.001), avoiding contact with the outer surface of clothing while removing (*p*<0.001) were less considered in the case group than control group. The results also showed that in 4.8% of the case group “touch of face, eyes, nose and mouth” occurred during the patient transfer, but this percentage for the control group was 0.7%; although this item was not significantly different in the two (*p*=0.166).

**Table 2:** PPE use precautions and self-care implementation in dealing with COVID-19 patients

**Variables**	**COVID-19 patient**	**PPE** ^i^	**Case group**	**Control group**	***p*** **-value** ^a^
			**Number (%)**		
Type of used face mask before entering the place where had a ...	Suspected	S^b^	42 (63.6)	97 (65.5)	0.865
		N^c^	10 (15.2)	29 (19.6)	
		S & N	10 (15.2)	21 (14.2)	
		E^d^	0 (0.0)	1 (0.7)	
	Confirmed	S	13 (19.7)	13 (8.8)	0.031
		N	35 (53.0)	109 (73.6)	
		S & N	14 (21.2)	25 (16.9)	
		E	0 (0.0)	1 (0.7)	
Type of used cloth before entering the place where had a ...	Suspected	U^e^	45 (68.2)	129 (87.2)	0.016
		I^f^	5 (7.6)	3 (2.0)	
		G^g^	8 (12.1)	15 (10.1)	
		NW^h^	2 (3.0)	0 (0.0)	
	Confirmed	U	11 (16.7)	2 (1.4)	<0.001
		I	12 (18.2)	104 (70.3)	
		G	7 (10.6)	0 (0.0)	
		NW	26 (39.4)	39 (26.4)	
		I-NW	1 (1.5)	0 (0.0)	
		G-NW	3 (4.5)	0 (0.0)	
		I-G	1 (1.5)	0 (0.0)	
General precautions of daily life		Yes	60 (90.9)	117 (79.1)	0.552
		No	0 (0.0)	3 (2.0)	

^a^ *p*-value refers to the relationship of each variable with two groups. Data was described using frequency(percent) or mean±SD. ^b^S: Surgical Mask; ^c^N: N95 Mask; ^d^E: Elastomeric Respirators; ^e^U: Uniform; ^f^I: Disposable, one-piece, waterproof protective cloth; ^g^G: Gown; ^h^NW: Disposable, one-piece, non-waterproof protective cloth; ^i^PPE: Personal Protection Equipment.

## Discussion

In this study we assessed the possible factors associated with the increasing of the risk of COVID-19 among EMTs and the results showed that those who were using surgical mask and NW cloths (disposable, one-piece, non-waterproof protective cloth) were more frequently affected by COVID-19 than those EMTs who used the other types. When it comes to the using PPE precautions, considerable number of affected EMTs were reported that despite receiving the instructions, they still used personal items such as watches, did not cover the hair with a medical hat, and did not perform the seal check; And also they did not pay attention to not touching the surface of PPE during doffing it. More importantly we found that when two EMTs involved directly in taking care of patients and those who worked with a confirmed case teammate, more frequently affected. On the other hand, the number of missions and the amount of working time during the last two months are not correlated with such risk.

To choose the best PPE, the infection control organizations recommended choosing after evaluating the risk level of exposure [7]. PPE should provide appropriate protection to the mode of viral transmission and be simple to use, easy to remove without contaminating the users, and ensure the use of PPE in a correct order, and also there should be a checklist regarding donning and doffing of PPE. All HCWs were recommended completing a checklist at the end of day at work and the data should be reviewed by the supervisors [4, 16]. Behavioral factors such as cough etiquette and adherence to the global health guidelines should also be considered during the management of a transmission [6]. The WHO recommended infection control strategies in the health care settings such as isolation of suspected and confirmed cases, applying standard precautions, and implementation of environmental controls [17].

A study compared the efficacy of different kinds of mask and the results showed that the surgical masks are low efficient in protecting 10-80 nm sized particles and the N95/FFP2 masks have at least 95% efficacy against 0.1–0.3 µm sized particles (size of droplet produced by coughing or sneezing is less than 5 µm) [18`]. The surgical mask have 80% protection against droplet particles and the N95 have at least 95% protection against aerosols particles which showed that using the N95 mask decreased the prevalence of contaminated HCWs significantly [4, 7, 19]. There are controversies in terms of mask type used. For example, a study in Canada, conducted by Bartoszko *et al*., showed that the efficacy of the medical and N95 masks had no significant difference among HCWs [20]. A case control study conducted on HCWs of five different hospitals in Hong Kong in 2004 reported that 72 HCWs were infected by SARS in the time of the study and wearing N95 or surgical masks had no role in this regard [16]. On the other hand, along with the results of our study, Seto *et al*., reported that the risk of SARS infection significantly decreased by using the N95 mask comparing to the surgical mask; However, because of its difficulties, the N95 mask was used less than the other mask types [21]. However, a study stated that correct use of surgical mask is more effective than wrong applying the N95 mask [7]. The failure rates of more than 5% in the use of N95 mask may occur due to the inappropriate use of mask like not fitting on the face [4]. Regardless of the quality of the filter materials, FFP-type masks may not provide reliable protection if the mask does not fit tightly. A study stated that the correct use of a surgical mask is more effective than wrong applying a N95 mask [7]. The N95 mask is a filtering, negative-pressure face-piece respirator, and its performance is highly dependent on a tight face seal [22, 23]; Unfortunately, the case group in our study had a significantly lower percentage of participants who performed seal check.

Most of the studies mentioned that education on donning, doffing, and other strategies on using PPE, were as important as adequate PPE supplies and could obviously decrease the cross-contamination. So training about the presentation of SARS disease, the routes of transmission, the appropriate use of PPE, and environmental factors became mandatory in a lot of hospitals [16-18, 24].

In this study, we found that the higher percentage of the case group’s participants significantly used accessories such as a watch, covered their hair with a medical hat, touched the outer surface of PPE during doffing, and etc. On the other hand, they were better in the PPE doffing in the correct order, adhering to precautions during the patient transfer, following the precautions while performing an airway management procedure. Our study showed that in cases where both of the EMTs were directly involved, the chance of being infected was increased. So in each mission only one EMT was directly in contact with the patient and evaluated him/her, and for the next mission the other EMT was directly in contact with the patient, by this way the frequency of exposure will be reduced.

On the other hand, due to the lack of equipment during large pandemics, the involvement of more people in providing direct patient care requires more PPE which lead to shortage of PPE in the system. Given that the history of the disease was higher in infected people than in non-infected ones, this difference was not statistically significant, which may be due to the low power of the study; Though all infected individuals were included in the study by the time of performing the project. It is better to identify the high-risk personnel during a pandemic and not assigning such missions to them as much as possible. On the other hand, the presence of a teammate before confirmation and quarantine increases the chance of infection by 4.98 times. Screening is very important in identifying the suspicious people.

We found that the type of used face masks (the surgical mask) and the type of used cloths (using NW) are correlated with the higher risk of COVID-19 in EMTs, in comparison with that of using other type of PPE. Improper use of PPE (did not cover the hair, did not check the mask seal, did not touching the surface of PPE during doffing) was also an important factor correlated with increasing the risk of COVID-19 infection. Eventually, we found that when two EMTs were involved directly in taking care of the patients, and those who worked with a confirmed case teammate, more frequently affected.

## Limitations

Due to the fact that the project was based on self-expression and retrospect, there is a possibility of bias on the part of the participants. As in this study, there is a possibility that there is a re-call bias. The participants in the control group did not do any diagnostic tests to check their health and because of the high prevalence of asymptomatic cases of COVID-19 reported in other studies, this may overestimate the efficacy of PPE and precautions (24). 

## Abbreviations

Emergency medical technicians (EMTs); coronavirus disease-19 (COVID-19); personal protective equipment (PPE); health care workers (HCWs); World Health Organization (WHO); reverse transcriptase polymerase chain reaction (RT-PCR); computed tomography (CT); S (the surgical masks); N (the N95 masks); E (the elastomeric respirator); U (uniform), I (disposable, one-piece, waterproof protective cloth); G (gown); NW (disposable, one-piece, non-waterproof protective cloth);

## Ethics approval and consent to participate:

The protocol of the study was approved by the ethics committee of Vice-Chancellor in Research Affairs - Tehran University of Medical Sciences and the code IR.TUMS.VCR.REC.1399.323 has been assigned to it. To maintain the principles of confidentiality, all information was analyzed and reported anonymously. Participants entered the study with informed consent.

## Availability of data and materials:

The dataset has been presented within the additional supporting files submitted in journal website.

## Consent for publication:

All the authors present their consent for publication of the paper.

## Funding:

This study was funded with a grant from Tehran EMS Center. The project was commissioned by the organization and the cost was paid to the contractor.
